# Bcl-2 Inhibition to Overcome Resistance to Chemo- and Immunotherapy

**DOI:** 10.3390/ijms19123950

**Published:** 2018-12-08

**Authors:** Marilina García-Aranda, Elisabet Pérez-Ruiz, Maximino Redondo

**Affiliations:** 1 Research Unit, REDISSEC, Hospital Costa del Sol, Autovía A-7, km 187, 29603 Marbella, Málaga, Spain; marilina@hcs.es; 2 Oncology Department, Hospital Costa del Sol, Autovía A-7, km 187, 29603 Marbella, Málaga, Spain; eliperu@gmail.es; 3 Research Unit, REDISSEC, Hospital Costa del Sol, Universidad de Málaga, Autovía A-7 km 187, 29603 Marbella, Málaga, Spain

**Keywords:** Bcl-2, resistance, apoptosis, inhibition, cancer, chemotherapy, immunotherapy

## Abstract

According to the World Health Organization (WHO), cancer is a leading cause of death worldwide. The identification of novel targets for cancer treatment is an area of intense work that has led Bcl-2 over-expression to be proposed as one of the hallmarks of cancer and Bcl-2 inhibition as a promising strategy for cancer treatment. In this review, we describe the different pathways related to programmed cell death, the role of Bcl-2 family members in apoptosis resistance to anti-cancer treatments, and the potential utility of Bcl-2 inhibitors to overcome resistance to chemo- and immunotherapy.

## 1. Introduction

### 1.1. Current Overview of Cancer Therapeutics

Cancer is a multifactorial disease involving both genetic and environmental factors that has become one of the main health issues around the world. The figures, only for the year 2012, include 14.1 million of newly diagnosed cases and the death of 8.2 million people worldwide [1]. Despite these figures, the great advances achieved in recent years in the field of cancer prevention and treatment have allowed a decrease in mortality trends [1]. The implementation of screening programs, the improvement of diagnostic and surgical techniques, as well as the development of new treatment strategies have been great allies in the search for a cure for oncologic patients.

However, and given that the finding of a unique cure for this complex disease does not seem close, current efforts in the battle against cancer are being focused on turning this disease into a chronic disease such as diabetes or acquired immune deficiency syndrome (AIDS), a goal that seems much closer since in this same year 2012, 32.6 million people were living with cancer (5-years observed survival after cancer diagnosis) [1].

**Figure 1:**
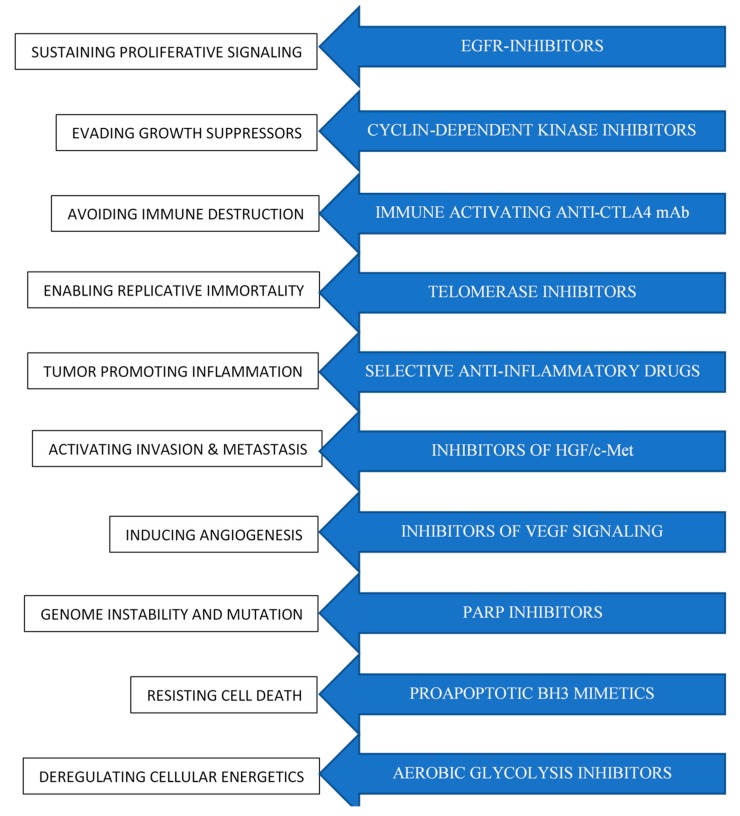
Chemotherapeutic agents designed to target different hallmarks of cancer [2]. Both cytotoxic and cytostatic agents are able to induce cell death by damaging cells at different levels.

Classically, malignancies have been classified on a histological basis (carcinoma, sarcoma, myeloma, leukemia, lymphoma, mixed) or attending to the original location where the tumor was developed (breast, lung, colorectal, prostate, stomach, etc.) and, in most cases, have been mainly treated with surgery as first-line therapy. Given that in its simplest form cancer is a disease caused by the uncontrolled division of abnormal cells, early cancer treatments were based on the use of cytotoxic drugs and radiotherapy that, preferentially, but not exclusively, cause actively proliferating cells’ death. Over time, treatments included the use of cytostatic drugs that worked by interrupting chemical processes involved in tumorigenesis and tumor progression. Both cytotoxic and cytostatic agents are able to induce cell death by damaging cells at different levels (Figure 1) (Table 1).

Chemotherapeutic agents have had a great impact in cancer treatment. However, since their targets are also present in normal cells that divide rapidly like those in bone marrow or intestinal lining tissues, both cytotoxic and cytostatic drugs cause many toxicities and a series of negative side-effects such as loss of hair, pain, vomiting, fatigue, blood clotting problems or a depressed immune system which impedes, in many cases, an appropriate drug dosage and requires the search of additional treatment alternatives.

Studies based on tumor molecular characteristics have allowed the elaboration of new classifications based on the expression profile of affected oncogene or tumor suppressor genes and have proved to be a useful tool for cancer definition and also for the development of novel targeted treatments, including those based on immunotherapy, with less secondary effects than conventional agents that lack tumor selectivity. 

A critical feature of advanced tumors is their capability to evade adaptive immune responses [3]. For this reason, the goal of immunotherapy is to take advantage of immune and inflammatory responses induced by malignancies to enhance the specificity and long-term memory of the adaptive immune system against tumor cells. The multiple approaches developed in recent years to achieve this objective include the application of exogenous cytokines, or other substances that increase the presence of tumor-specific T cells, the transfer of tumor-specific immune effector cells or the inhibition of tumor-induced immune-suppressive mechanisms with immune checkpoint inhibitors and agonists of co-stimulatory receptors [4] among others [5]. (Table 2). 

The significant progress achieved in this field has established cancer immunotherapies as a valuable tool to defeat different types of cancer, including melanoma, renal cell carcinoma or hematologic malignancies [6]. As a result, multiple cancer immunotherapies have demonstrated their efficacy and many of them have already been approved for cancer treatment [5,6]. Despite this progress, some cancers and some patients will show a limited clinical response to such therapies or will reveal a series of adverse effects and toxicities [6] which justify further research in this respect. 

### 1.2. Programmed Cell Death

Because apoptosis evasion is one of the hallmarks of cancer [7], most anti-cancer treatments are designed to exert their activity by the induction of tumoral programmed cell death, or apoptosis, and/or the activation of related cell death networks [8]. 

Apoptosis, which is a highly conserved and regulated process that allows the natural elimination of aged or damaged cells from the body, involves energy-dependent signaling pathways that can be activated through a wide variety of stimuli and conditions [9]. Apoptosis deregulation has been related to cancer, autoimmune disorders or degenerative diseases among others [9,10]. 

There are two major apoptosis pathways: the extrinsic and the intrinsic pathways, both of them converging on an irreversible cell death mainly mediated by the activation and coordination of proteolytic caspases (cysteine-aspartic proteases). (Table 3).

#### 1.2.1. The Extrinsic Pathway

The extrinsic pathway is mediated by plasma-membrane death receptors and ligands [16] or by the action of cytotoxic granules [17]. 

##### Death-receptor-mediated apoptosis

During this extrinsic apoptosis pathway, the binding of a death receptor with its ligand results in the activation of a death-inducing signaling which is mainly regulated by caspase-8 inhibitory protein FLIP [18]. Tumor necrosis factor (TNF)-receptor (TNF-R) and TNF-ligand (TNF-L) super-families are the major extrinsic apoptosis signaling mediators in humans. 

Members of the TNF-R superfamily are type I transmembrane proteins consisting of an ectodomain (an elongated cysteine-rich domain formed of three disulfide bonds surrounding a CXXCXXC core motif [19]), a transmembrane domain and an intracellular domain responsible for the activation of signal transduction pathways inside the cell [20]. The number of these cysteine-rich domains is variable among TNF-R members [19]. 

According to their downstream interaction partners, TNF-R members can be classified into three groups:

1. TNFR1 (also known as death receptors or p55/p60 [21]): a death-domain-containing-protein in the intracellular portion which activates apoptosis via activation of intracellular death-inducing signaling complex (DISC) proteins [21], including Fas-associated proteins with death domain (FADD), TNFR1-associated death domain protein (TRADD) and other death domain-binding partners [20]. Most cells express constitutive but low levels of TNFR1 [21].
2. TNFR2 (also known as TNFR-associated factor (TRAF)-interacting receptors or p75/p80) [21], that interact with members of the TRAF family [20]. Only some cells express detectable surface receptors from this group [21].
3. Decoy receptors (DcR) with no intracellular interacting partners that act as TNF superfamily ligand inhibitors [20].

Most TNF-L-superfamily members, like FasL (CD95L) or TRAIL (APO-2L), are type II transmembrane proteins [20] expressed on the surface of activated monocytes/macrophages, activated NK and T cells and other non-immune cells such as endothelial cells and fibroblasts [21]. These transmembrane receptors can be cleaved by the TNF*alpha*-converting enzyme metalloprotease and transformed into a trimeric soluble cytokine [21] with the ability to act at distant physiological sites [21]. Although both soluble and membrane TNFLs can bind to TNFRs, the membrane ligands are more potent ligands for TNFR2 [21] and also produce more effective signals than the soluble ones [20]. In this regard, although some TNF-L only bind to one specific TNF-R, most of the TNF-L can bind to more than one receptor, resulting in a complex signaling transduction [20] in which at least 19 TNF-L and 29 TNF-R members have been implied to date [20]. These biological interactions between each ligand-receptor pair are being considered as systems responsible for numerous, complex and even divergent signaling pathways that can lead to both cell death or cell activation [21].

During death-receptor-mediated apoptosis, the activation of death receptors leads to the formation of DISC (death-inducing signaling complex), a multi-protein complex containing death receptors, adaptor proteins, caspase-8 and caspase-10 [22] that initiates a downstream signal cascade resulting in cell death. Recent studies also show that in most cells, after death receptor signaling, caspase-8 can directly activate downstream caspases and cell death [23].

##### The cytotoxic granule-mediated cell death

This extrinsic death pathway is the mechanism by which cytotoxic T lymphocytes (T cells) and natural killers (NK) eliminate targeted cells (harmful allogenic, infected or tumor cells) by means of the release of cytotoxic granules content into the immunological synapse between the cytotoxic cell and the targeted cell [17,24]. These lytic granules contain granzyme serine esterases (A, B, H, K, M), the lethal pore-forming perforin as well as other proteases [17,24]. 

Once in the target cell cytoplasm, granzymes can activate the intrinsic apoptosis pathway by directly inducing the mitochondrial outer membrane permeabilization [17]. Granzymes can also initiate cell death programs via proteolytic cleavage of substrate proteins such as initiators and executioner procaspases [17] as well as parallel, caspase-independent cell death pathways via the cleavage of proteins responsible of DNA damage and fragmentation, among other nuclear proteins [9,17], or the split of structural, cytoskeletal, translation or mitosis proteins [17]. 

#### 1.2.2. The Intrinsic Pathway

The other major apoptosis pathway, the intrinsic pathway, can be activated by both exogenous and endogenous stimuli [12], and includes the mitochondrial, the lysosomal and the *Trp53* tumor suppressor gene product (p53)-induced protein with a death domain (PIDD)-osome death pathway [17] among others. (Table 4).

Mitochondria is the master key of the intrinsic pathway. During the final execution pathway outer mitochondrial membranes become permeable to internal cytochrome and other mitochondrial intermembrane proteins, such as Smac/DIABLO, that lead to caspase activation [23]. Cytochrome-c is an indispensable component of mitochondrial membrane respiratory electron transport chain whose release into the cytosol disables energy production as well as APAF-1 (apoptotic protease activating factor 1) protein activation [28,29]. In the presence of dATP and cytochrome c, activated APAF-1, which contains a N-terminal caspase recruitment domain, binds two pro-caspase-9 molecules together to compose the apoptosome which subsequently splits and activates the effector protease caspases-3 and -7 [30]. Caspases-3 and -7 can cleave the initiator caspase 8 and activate a positive feedback loop for this cascade [30]. 

### 1.3. Resistance to Chemo- or Immunotherapy-Induced Apoptosis

Both chemo- and immunotherapies can directly or indirectly activate the cellular apoptosis machinery. For this reason, tumor cell sensitivity to anti-cancer agents depends on the level of expression of anti-apoptotic proteins [31] as well as on their ability to activate apoptotic pathways in response to extrinsic and intrinsic death signals [17]. Similarly, tumor sensitivity to immune-therapies would also rely on HLA (human leukocyte antigen) class I and II antigens expression and apoptosis-regulating proteins expression [32].

Genomic instability along with natural selection provoked by the selective pressure caused by anti-cancer treatments can promote the emergence of resistant cell populations within tumor cells. This phenomenon, which implies the lack of response and the impossibility to eliminate all cancer cells in a tumor mass, is due to host and tumoral-related factors [33], in which a failure in activating apoptosis plays a key role [8]. 

To overcome treatment resistance, disease recurrence and the emergence a mortal metastatic disease, current strategies normally require the use of combined therapies, with minimally overlapping toxicities to allow maximal dosages and narrowest cycle interval [34], targeting alternate pathways to cell death. However, there is still a high probability for cancer cells to develop multidrug resistance, which has been described as the single most common reason for chemotherapy discontinuation [35].

For these reasons, the mechanisms by which tumor cells manage to become resistant and evade the immune system and chemotherapy-induced cell-death are currently under an intense study in order to develop new strategies that bypass altered pathways and activate alternate routes causing cell death. In this regard, different studies have identified different enzymes involved in apoptosis pathways, highlighting the role of Bcl-2 proteins.

## 2. BCL-2 as a Target for Cancer Treatment

### 2.1. The Bcl-2 Family

An increased understanding of the molecular pathways underlying apoptosis has been one of the main goals in recent cancer research, which has led to the development of new targeted approaches for cancer treatment and to overcome tumor cell resistance.

As a result of such investigations the B-cell lymphoma-2 (Bcl-2) proteins, a protein family that participated in the regulation of many vital cellular functions [36], but also acted as master regulators of apoptosis, were identified three decades ago. This discovery not only revolutionized the knowledge about the role of programmed cell death during normal tissue development and homeostasis but, also, during tumor progression, tumor regression [30,37] and induced cell death [38].

The Bcl-2 protein-family comprises three subfamilies, with both pro- and anti-apoptotic roles, sharing a similar three-dimensional structure [39] and carrying one and four highly evolutionary conserved BCL-2 *alpha*-helices homology (BH) domains (BH1, BH2, BH3, BH4) [40]. (Table 5). 

**Figure 2:**
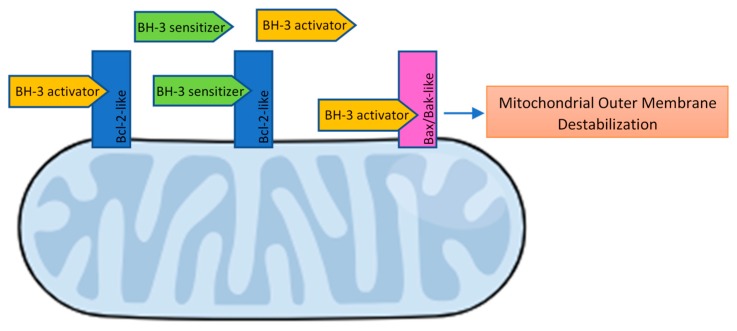
BH3-only effectors and sensitizers mechanism of action. Antiapoptotic Bcl-2-like proteins can sequester and inactivate BH3-activator and sensitizer proteins, preventing apoptosis. Excess BH3-sensitizer proteins prevent the sequestration of BH3-activators by Bcl-2-like proteins, allowing them to directly interact with and activate Bax/Bak-like apoptosis effectors.

**Figure 3:**
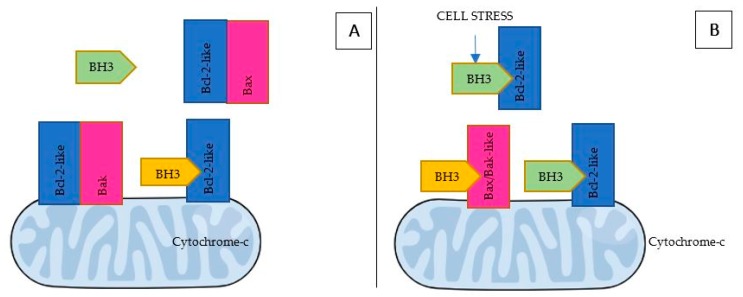
(**A**) Pro-apoptotic BH3 (only) or Bax/Bak-like proteins’ sequestration by anti-apoptotic Bcl-2-like proteins leads to cell survival. (**B**) BH3 (only)-proteins respond to cell stress provoked by chemo-therapeutic agents (Table 1) by binding to and inactivating anti-apoptotic Bcl-2-like proteins. BH3-only proteins can also engage Bax/Bak, causing their conformational transformation, activation and mitochondrial outer membrane destabilization [90].

The complex framework of BH3-mediated interactions between the pro- and anti-apoptotic Bcl2-family members has been widely covered by academic articles and reviews and is briefly summarized below:

- Bcl-2 apoptosis effectors (Bax, Bak, Bok), whose oligomerization plays a central role during mitochondrial outer membrane destabilization, apoptogenic proteins (cytochrome c, SMAC, Htra2, etc.) release and caspase activation [89], are tightly controlled by other Bcl-2 proteins [18]. Recent studies also propose a “membrane-induced spontaneous Bax/Bak activation model” in which the outer mitochondrial membrane plays a central role in Bax/Bak activation regardless of the presence of other Bcl-2 proteins [90].
- BH3 (only) pro-apoptotic initiators [91] are able to respond to cellular stress by, directly or indirectly, activating the apoptosis effector (or executioner) members. These proteins are tightly regulated on the transcriptional and post-transcriptional level and can act as apoptosis sensitizers or activators [18]. BH3-activators (Bid, Bim, Puma, Bmf) bind to both the pro-apoptotic and the anti-apoptotic Bcl-2 multi-region members whilst BH3-sensitizers (Bad, Noxa, Hrk, Bik) unbind activator BH3-proteins and bind to the anti-apoptotic members, boosting mitochondrial outer membrane permeabilization [41,90]. Excess sensitizer proteins prevent activator-proteins sequestration by anti-apoptotic homologues, allowing them to directly interact with and activate the apoptosis effectors [67] (Figure 2). Both the amphipathic *alpha*-helical BH3-domain and the presence of the apoptotic effector members, are essential for BH3-only proteins death function [92].
- Bcl-2 anti-apoptotic members (Bcl-2-like proteins: Bcl-2, Bcl-xL, Bcl-W, Mcl-1, Bcl-B, Bcl2A1) [91], act directly preventing effectors oligomerization or indirectly sequestering and inactivating BH3-only proteins to prevent the effectors’ activation. Thus, the anti-apoptotic members can block both the BH3 (only) as well as the effector members [92] (Figure 3).

**Figure 4:**
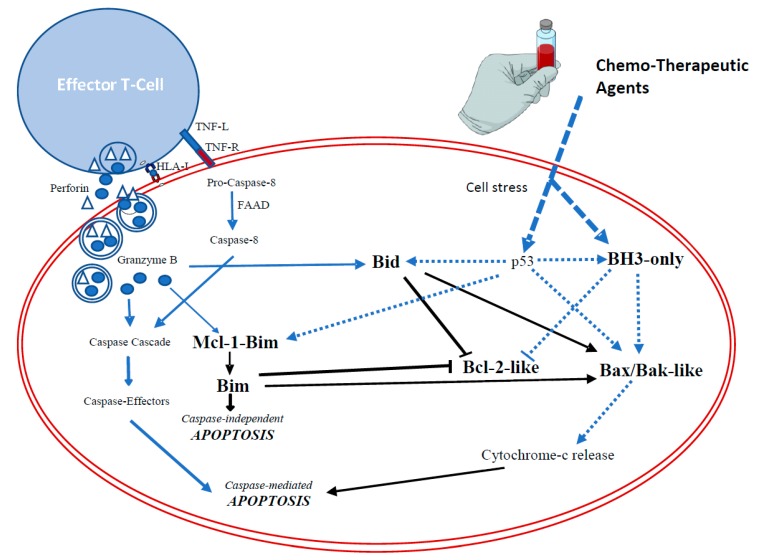
Apoptosis pathways mediated by chemo- and immunotherapy. Both chemo- and immunotherapy converge on common pathways (black lines) in which pro-apoptotic Bid and Bim play a key role as connecting elements. **Cell death induced by chemotherapy (blue dotted line):** Cell stress caused by chemotherapy entails the activation of p53 and BH3-only-proteins. As a result, proapoptotic Bid, Bim and Bax/Bak-like proteins are activated and anti-apoptotic Bcl-2-like proteins inhibited. **Cell death induced by immunotherapy (blue solid line):** T lymphocytes can trigger cell death by directly activating caspase cascade and by activating caspase-independent apoptosis mediated by Bim as well. This cytotoxic effect is also enhanced by activating pro-apoptotic Bid.

The whole family plays a central role in the regulation of apoptosis machinery and its correct functioning is a key element in the effectiveness of current anti-cancer treatments [39] (Figure 3) (Figure 4). Indeed, Bcl-2-like proteins over-expression [37,93] and/or mutations that affect BH3-only proteins induction [91] is related to enhanced apoptosis evasion [89] and resistance to chemo-therapeutic agents or immune-surveillance [39,89,94,95,96].

Provided that the ratio between pro-apoptotic (Bax/Bak-like) and anti-apoptotic (Bcl-2-like) proteins will determine if the cell dies or survives [97,98], strategies aimed to increase this ratio appear as a rational approach to enhance the efficacy of anti-cancer treatments [98,99,100]. In this regard, apoptosis evasion via Bcl-2-like proteins’ over-expression has recently been proposed as a hallmark of cancer [37] and hence, as a promising target for new treatment development.

### 2.2. BCL-2 Inhibitors to Overcome Resistance to Anti-Cancer Treatments

The study of mechanisms related to resistance against anticancer treatments is a field of intense research nowadays. As a result of this effort, the loss of HLA class I and II [32,101] and alterations such as anti-apoptotic genes’ over-expression or pro-apoptotic genes’ silencing, tumor suppressor gene p53 down-regulation [39] or aberrant protein kinase pathways regulation [39,102] stand out as the main causes of apoptosis resistance against immune response or chemotherapeutic agents.

Recent studies have suggested that, since with Bcl-2, or related anti-apoptotic proteins, over-expression supports apoptosis evasion and promotes cell survival, cancer cells can become Bcl-2 dependent to continue surviving under the selective pressure of an aggressive environment caused by immune system or chemotherapeutic agents. Under these circumstances, tumor cells become “Bcl-2 addicted” and at the same time more susceptible to Bcl-2 inhibitors [89]. In this context, it is interesting to note that blood cancers are usually depend on Bcl-2 while solid tumors appear to be more dependent on other anti-apoptotic proteins such as Bcl-xL or Mcl-1 [103]. 

**Figure 5:**
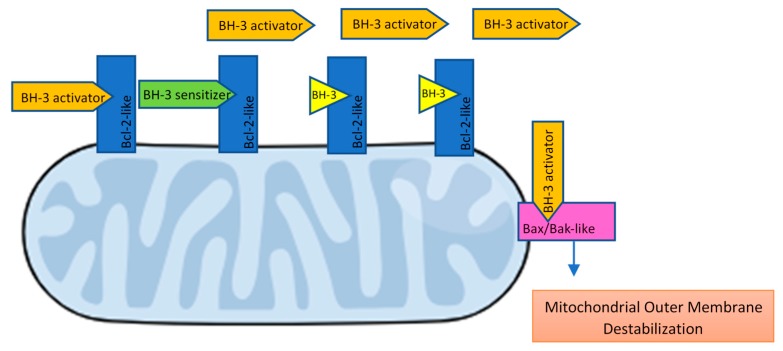
BH3-mimicking and apoptosis induction BH-3-mimicking peptides bind to anti-apoptotic Bcl-2-like proteins as competitive inhibitors, allowing BH-3-activators release and pro-apoptotic Bax/Bak-like proteins activation leading to cell death.

Strategies to inhibit Bcl-2-like proteins include the use of antisense oligonucleotides, antibodies, peptides or small molecule inhibitors, each of which with their pros and cons have recently been fully reviewed in literature [39,92,104]. Among these strategies, due to their smaller size and high specificity to bind to the three-dimensional structurally conserved hydrophobic cleft present on anti-apoptotic Bcl-2 family proteins [92], the use of BH3-domain-mimicking peptides looks like the most promising alternative [39] and has led to the development of a series of small molecules that bind to the hydrophobic cleft of anti-apoptotic Bcl2-proteins as competitive inhibitors [104] that are currently under different clinical and preclinical stages of development for cancer treatment [39,89,91,105]. (Figure 5) (Table 6 and Table 7).

Venclexta (venetoclax), which has recently been approved by the FDA for the treatment of lymphocytic leukemia patients [106], has also proven to be effective against relapsed and refractory chronic lymphocytic leukemia, including disease resistant to DNA-damaging chemotherapy with poor prognosis features [91]. Apart from Venclexta, many other Bcl-2 inhibitors have already proven in in vitro and in vivo studies to be effective in inducing cell apoptosis [39] (Table 6) [104] even in p53-defective tumors, since BH3 mimetics act downstream this protein [91]. However, despite BH3 favorable clinical responses [89] as monotherapy or in combination with other approved or experimental anticancer drugs [104] and immunotherapies [94,107] in Bcl-2 over-expressing cells [94], studies carried out in in vivo models show that these small molecule inhibitors would also interfere with T-cell immune response, due to the higher sensitivity of early-activated T cells to Bcl-2 inhibitors, which would require the administration of Bcl-2 inhibitors and vaccines at different times [108,109].

Even though the number and the advanced development stage of studies evaluating the therapeutic potential of Bcl-2 inhibitors, publications about the utility of these molecules to reverse the resistance to tumor cells are scarce. Nonetheless, provided that Bcl-2, Mcl-1, Bcl-xL or Bcl2A1 over-expression is related to acquired chemo-resistance [110] and that the inhibition of Bcl-2-like proteins increases the effectivity of anti-cancer drugs [31], eliminating cancer stem cells [111] as well as apoptosis-resistant cells [39,92,112], additional studies targeting these proteins to overcome resistance against anti-cancer treatments are justified. In this context, considering the conserved three-dimensional structure and BH domains of Bcl-2 family members, there is still a need to continue investigating the role of each amino-acid sequence in determining the opposing roles of both pro- and anti-apoptotic Bcl-2 members [39] in order to develop new drugs with an increased efficiency and efficacy or with the ability to overcome acquired resistance due to mutations in these domains.

On the other hand, it is worth noting that malignancies with an altered HLA system would not achieve an optimal response to these therapies (Figure 4) and would require previous studies for patients’ selection. Similar to such cases, since apoptosis resistance related to an imbalance between Bcl-2-family members can be caused by an increased expression of anti-apoptotic Bcl-2 proteins, the loss of function of BH3-only activator proteins or mutations causing effector Bcl-2 proteins loss or inactivation [92], tumors with altered effector or activator proteins will not respond to therapies based on the use of BH3 mimetics and would justify the use of BH3 profiling techniques [39,92,100] or systematic mapping of Bcl-2 gene dependencies [113] to identify patients that would better respond to the treatment. 

In this context, provided that the switch from survival to apoptosis occurs when the concentration of BH3-only proteins is sufficient to both neutralize Bcl-2-like and activate Bax/Bak-like proteins [91], strategies aimed to restore the normal expression of BH3 (only) proteins in general, and Bid/Bim in particular as connecting elements between chemo- and immunotherapy- mediated cell death (Figure 4), would be valuable tools to overcome acquired resistance [74].

Genomic instability together with improved cell survival over time, increases the probability that tumor cells acquire new mutations and the subsequent expansion of populations resistant to BH3 mimetic inhibitors [114], a phenomenon that has already been demonstrated in in vitro studies and that would require the use of combined therapies with different Bcl-2-like inhibitors [113,115,116]. Accordingly, although the use of BH3-mimetics such as monotherapy have a limited efficacy against epithelial cancers such as ovarian, pancreatic or breast cancers [92], their use in combination with non-conventional [117,118,119,120] and conventional anti-cancer agents in the treatment of different malignancies including melanoma, glioma, multiple myeloma or breast and pancreatic cancer have shown proven benefits [92,121]. The positive effect of BH3-mimetics in re-sensitizing tumor cells to conventional treatments also supports the use of these small peptides in combination therapies [92]. However, an important consideration to mention here is that the effect of BH3 mimicking peptides could have adverse effects such as tumor lysis syndrome, neutropenia [91] or calcium-signaling dysregulation not only in cancer cells but also in healthy cells [122], which may become a limitation for this type of therapeutic regimens.

Finally, it is worth noting the increasing relevance of studies about new inhibitors targeting the BH4 domain of Bcl-2-like proteins, which have already shown their promising ability to increase the action of chemotherapeutic drugs both in vivo and in vitro [123]. In this context, the important effect of different micro-RNA on Bcl-2-like proteins regulation in cancer cells in general [124,125,126] and controlling the balance between the pro-survival and the pro-apoptotic Bcl-2 proteins in particular [127], could help expand our current knowledge about the role of Bcl-2 proteins in drug resistance [128,129] and might also become an innovative approach for improving sensitivity against chemo- and immunotherapy [129].

## 3. Conclusions

Studies published to date show the clear implication of aberrant Bcl-2 proteins’ expression in carcinogenesis and apoptosis resistance after chemo- or immuno-therapies, which supports the development of Bcl-2-targeting strategies to overcome resistance and improve a patient’s clinical management and survival. In this regard, the evolving field of bioinformatics and personalized medicine approaches coupled with molecular biology studies focused on Bcl-2-related pathways are portrayed as tools with greater projection.

## Figures and Tables

**Table 1:** Chemotherapeutic agents: classification and mechanisms of action.

	Mechanism of Action	Site of Action	Examples
Antimetabolites	Interfere with DNA/RNA synthesis by inhibiting purine ring synthesis, ribonucleotide reductase or DNA monomers synthesis normally causing cell death during the S phase of cell growth.	Purines and pyrimidines synthesis	Thioguanine Mercaptopurine Methotrexenate
		Ribonucleotides	Hydroxycarbamide
		*DNA* monomers	Methotrexate 5-Fluorouracil
		*DNA* synthesis	Cytarabine Bleomycin
Intercalating agents	Interfere with DNA/RNA synthesis, preventing cell duplication		Topoisomerase inhibitors (Etoposide Topotecan, Irinotecan) Antibiotics (anthracyclines, Chromomycin, Doxorubicin)
Cross-linking agents	Cross-link two DNA bases together preventing DNA from being separated during DNA synthesis or transcription. Nucleotide mispairing, leading to mutations.		Alkylating agents Nitrosoureas Platinum-based coordination complexes
Enzyme	Protein synthesis inhibition, leading to cell death by apoptosis	Protein synthesis	L-Asparaginase
Microtubule damaging agents	Anti-mitotic agents that inhibit cell proliferation by disrupting the normal function of the mitotic spindle.	Microtubules	Taxanes Vinca alkaloids Taxol Colchicine
Enzyme inhibitors	Interfere with normal cell metabolism, leading to cell death	Enzyme activity	Methotrexate Kinase inhibitors
Angiogenesis inhibitors	Inhibit endothelial cells’ proliferation and tumor growth	Angiogenesis	Endostatin

**Table 2:** Classification of current anticancer immunotherapies [5].

Classification	Overview	
Tumor-targeting immunotherapy	Naked monoclonal antibodies	Bind and alter the signaling pathways required by malignant cells’ survival or progression.
		Activate lethal receptors expressed on the surface of cancer cells.
		Opsonizing antibodies that bind to specific tumor-associated antigens.
	Conjugated monoclonal antibodies	Tumor antigen-associated antibodies coupled with toxins or radionuclides.
		Bi-specific T-cell engagers that enhance immune response.
Oncolytic viruses	Non-pathogenic viral strains that infect and directly or indirectly lead to cancer cells’ death.	
Anticancer vaccines	Peptide- and DNA-based vaccines to enhance the ability of resident antigen-presenting cells to present tumor-associated antigens, which activates the host immune system against tumor cells.	
	Isolation, ex vivo amplification/differentiation/activation and administration of dendritic cells which engages the host immune system against tumor cells.	
	Administration of immunomodulatory cytokines, generally as adjuvants for other anticancer treatments.	
	Administration of immunomodulatory antibodies such as checkpoint blockers or those that interact with soluble or cellular components of the immune system and activate the immune response.	
	Administration of inhibitors of immunosuppressive metabolism which alters cancer cells’ microenvironment with antineoplastic effects.	
	Pattern recognition receptors-agonists which activates signal transduction cascades with pro-inflammatory effects that include the activation and secretion of immunostimulatory cytokines, dendritic cells maturation and macrophages/natural-killer cells activation.	
	Immunogenic cell death inducers, such as some conventional chemotherapeutics, that stimulate the release of damage-associated molecular patterns by cancer cells, which enhances the activation and maturation of antigen-presenting cells.	
Adoptive cell immunotherapy	Collection, ex vivo selection/modification/expansion/activation and administration of circulating or tumor-infiltrating lymphocytes.	
	Administration of genetically modified T-cells with enhanced proliferative potential and persistence, unique antigen specificity or improved secretory profile, tumor-infiltrating capacity or cytotoxicity.	

**Table 3:** Caspases Classification.

Caspase group	Members	Overview
Initiator caspases	Caspase-2,-8,-9,-10	These caspases are at the top of the caspase signaling cascade, being responsible for executioner caspases proteolytic activation during apoptosis [11]. Initiator caspases are characterized by the presence of an extended N-terminal pro-domain essential for their function [11].
Effector or executioner caspases	Caspase-3,-6,-7	Effector caspases [9,12,13,14] induce biochemical and morphological changes in the cell such as chromatin condensation, DNA and nuclear fragmentation, cytoskeletal and nuclear protein degradation, crosslinking of proteins, formation of apoptotic bodies and expression of ligands for phagocytic cell receptors [9].
Inflammatory caspases	Caspase-1,-4,-5,-11	Key regulators of inflammation and cell death by inducing pyroptosis and the extracellular release of pro-inflammatory cytokines and danger signals [15].
Other caspases	Caspase-12 caspase-13 and caspase-14	These caspases are not well characterized and are still under study. Caspase-12 has a role during endoplasmic-specific apoptosis [9].

**Table 4:** Main apoptosis intrinsic pathways.

Pathway	Activation	Description
Mitochondrial pathway	As a response to different stress signals such as DNA damage, chemotherapeutic agents or ultraviolet (UV) light.	Triggers mitochondrial membrane destabilization and permeabilization, with the subsequent release of pro-apoptotic intermembrane itochondrial factors, such as the enzyme cytochrome C oxidase [12,17], which leads to apoptosome formation, caspase-9 activation [18] and cell death.
Lysosomal pathway	As a response to oxidative stress, death-receptors activation, viral proteins and other death stimuli [25].	Lysosomal membrane permeabilization triggers the release of cathepsins into the cytoplasm, outer mitochondria membrane destabilization and subsequent cytochrome-c release [17,26].
PIDD-osome pathway	As a response to DNA damage [27].	p53 tumor suppressor gene product (p53)-induced proteins with a death domain (PIDD) can activate caspase-2 and the caspase proteolytic cascade leading to the final execution pathway [27].
Endoplasmic reticulum pathway	Under study.	Sensitizes mitochondria to both extrinsic and intrinsic death signals as well as by initiating cell death signals [28].

**Table 5:** Members of the BCL-2 protein family.

BCL-2 Subfamily		Members	BH Domains	Overview
Anti-apoptotic		Bcl-2	BH1 BH2 BH3 BH4 TM	Bcl-2 is constitutively bound to mitochondrial and/or endoplasmic reticulum membranes and represents the main pro-survival member of the Bcl-2 family [41]. Bcl-2 can sequester activator and sensitizer BH3-only proteins and is also able to bind to the inositol trisphosphate receptors (InsP3R), membrane glycoprotein complexes acting as membrane calcium channels on the endoplasmic reticulum, inhibiting the initiation phase of calcium-mediated apoptosis [42]. Bcl-2 over-expression has been widely described in different types of malignancies and is related to tumor formation, progression, therapy resistance and poorer overall survival [39,43,44]. Although negative Bcl-2 expression has been proposed as a marker of good chemotherapy response in breast cancer patients [45], recent studies have shown Bcl-2 over-expression as a good prognosis factor in patients with different types of cancer including breast [46], colorectal, renal and advanced non-small cell lung cancer [47,48,49].
		B-cell lymphoma-extra large (Bcl-XL)	BH1 BH2 BH3 BH4 TM	Bcl-XL is a transmembrane protein localized in the outer mitochondrial and nucleus membranes, where it may bind to nuclear proteins and modulate transcription factors activity [36]. Bcl-XL can also sequester cytoplasmic p53, inhibiting cell death [50]. Bcl-XL, is usually over-expressed in different types of cancer and has been related to cancer cell growth, migration, invasion, maintenance of cancer stem cell phenotype, angiogenesis, enhanced aggressiveness [36] and apoptosis resistance [36,39].
		Bcl-2-like protein 2 (Bcl2l2, Bcl-W)	BH1 BH2 BH3 BH4 TM	The active Bcl-W isoform is loosely attached to mitochondria and can be neutralized by BH3 (only) proteins by enhancing the insertion of its C-terminal domain into the membrane [51]. Bcl-W over-expression has been related to different malignancies, including lymphoma, colorectal cancer and gastric cancer [52,53,54,55] and to a worse prognosis [54]. Bcl-W expression is regulated by MYC transcription factor through a specific microRNA [54] and its amount within the cell is modulated by the Akt serine-threonine kinase [56].
		Induced myeloid leukemia cell differentiation protein (Mcl-1)	BH1 BH2 BH3 BH4 TM	Mcl-1 binds to pro-apoptotic Bim [17], Bak and Bax [57] proteins to prevent apoptosis. Mcl-1-Bim complexes can be cleaved by granzyme B, allowing outer mitochondrial membrane permeabilization and apoptosis [17]. Mcl-1 is highly over-expressed in cancer cells [18] and has been related to chemotherapy-resistance [39].
		Bcl-2 related protein A1 (Bcl2A1), Bfl-1	BH1 BH2 BH3 BH4	In response to apoptotic stimuli, Bcl2A1 can translocate from the mitochondria or the cytoplasm to the nucleus [58] where its role remains unclear. Similarly to Mcl1, Bcl2A1 pro-survival ability is related to its association to pro-apoptotic BH3-only Bim, Bid and Puma proteins as well as to Bik, Hrk and Noxa [18]. Although Bcl2A1 is usually over-expressed in cancer cells [18] and contributes to the acquisition of tumor cell resistance against chemotherapy-induced apoptosis [58], the role of Bcl2A1 in both healthy and cancer cells is still under study [58]. Bcl2A1 is regulated at post-translational level by the proteasome and by transcription factors such as NFĸB [58] or retinoic acid [18].
		Bcl-B Bcl2l10	BH1 BH2 BH3 BH4 TM	Bcl-B binds to Bcl-2, bcl-XL and Bax, but not to Bak, and is able to suppress Bax-induced apoptosis in vitro [59]. Bcl-B is over-expressed in multiple-myeloma patients [60].
Pro-apoptotic	Effectors	Bcl-2-associated X protein (Bax)	BH1 BH2 BH3 TM	Along with Bak, Bax is one of the main apoptosis effectors. Bax exists as a free inactive cytosolic protein that responds to various stimuli exposing the BH3 domain to allow oligomerization [23] and then migrating and inserting into the mitochondria membrane, inducing the release of cytochrome-c [30]. Bax activity is mainly regulated by the cytosolic accumulation of the tumor suppressor protein p53 [61] as well as by other Bcl-2 family members [23].
		Bcl-2 homologous antagonist killer (Bak)	BH1 BH2 BH3 TM	Bak, is one of the main apoptosis effectors. After activation by stress signals, this integral mitochondrial membrane protein is activated by exposing the BH3 domain to allow oligomerization and outer mitochondrial membrane destabilization [23]. Bak can directly be activated by the tumor suppressor p53 by blocking the Mcl1 anti-apoptotic effect [62] and can also be regulated by other Bcl-2 family members [23].
		Bcl-2 related ovarian killer (Bok)	BH1 BH2 BH3 TM	Contrary to Bax or Bak, Bok is constitutively active and unresponsive to the inhibitory effects of Bcl-2 anti-apoptotic members [63], being able to trigger mitochondrial membrane permeabilization and apoptosis independently of Bax and Bak presence [63]. Bok activity, which is controlled by ubiquitylation and proteasomal degradation [63], is an essential mediator of p53-dependent apoptosis [64].
	Activators	BH3-interacting domain death agonist (Bid)	BH3	Bid responds to tumor suppressor p53, contributing to cell death as response to cell damage after chemotherapy [65,66]. On the other hand, Bid can also be cleaved and activated by granzyme B [17] as well as by Caspase-8 after death receptor signaling (Fas-ligation-mediated apoptosis). For these reasons, Bid has a key role as a connecting element between the intrinsic and the extrinsic apoptosis pathways [67]. After activation, Bid exposes the BH3 domain which allows its dimerization with apoptosis-effectors Bax, Bak and anti-apoptotic Bcl-2-like proteins [23], resulting in Bax and Bak activation and Bcl-2-like proteins inhibition and subsequent cell death. Once activated, Bid can also migrate from cytosol to mitochondria where it can directly promote the release of cytochrome c and other apoptogenic factors [17,68], amplifying caspase activation. Low Bid expression is related to resistance to chemotherapy [69] and TRAIL [70].
		Bcl-2-like protein 11 (Bim)	BH3 TM	Bim can appear associated to microtubules [67] or sequestered forming complexes with all pro-survival proteins [23]. These complexes can be disrupted by tumor suppressor p53 [71] as a response to cellular stress [23] and also by Granzyme B [17], allowing Bim activation and translocation to mitochondrial outer membrane to indirectly cause cell death by pro-apoptotic Bak/Bax activation [67,72,73]. Bim expression is regulated at different levels, and its abundance is controlled via the proteasome by protein kinases downstream growth factor receptor activation [67]. Bim has been reported to play a central role in regulation of tumorigenesis [74]. Indeed, Bim over-expression inhibits tumor growth and drug resistance [74], while Bim loss is associated with lymphadenopathy, autoimmunity [67] and tumor promotion [74].
		p53 upregulated modulator of apoptosis (Puma)	BH3	Similarly to Bid and Bim, Puma can directly bind and antagonize all pro-survival proteins [23,75] by directly or indirectly promoting cell death [75,76]. Puma, whose expression can be induced by nuclear p53 [50,76] after cellular stress or DNA damage [23,50,77], is able to displace cytoplasmic p53 from anti-apoptotic Bcl-xL, allowing p53 to induce cell death [50]. Puma expression can also be activated by transcription factors induced as a response to stimuli such as genotoxic stress, deregulated oncogene expression or toxins, being able to induce cell death in a p53-independent manner [75]. Puma, which is required by Bad and Noxa to induce cell death [73], can also directly activate pro-apoptotic Bax and Bak to promote mitochondrial cytochrome c release [73]. Aberrant Puma expression has been related to increased cancer risk development and therapeutic resistance [67,75].
		Bcl2 like 11, Bcl2 modifying factor (Bmf)	BH3	Similar to Bim, Bmf is bound to cytoskeletal structures [67,78]. After cellular damage or anoikis, Bmf is unleashed, being able to sequester pro-survival Bcl-2 proteins and promote cell death [78]. Bmf, which is widely expressed in lymphocytes and most hematopoietic tissues [79], is also expressed in different malignancies [80] including lung and breast [79]. Aberrant Bmf expression has been related to acquired resistance to chemotherapy [81].
	Sensitizers	Bcl-2-associated death promoter (Bad)	BH3	Bad promotes apoptosis by interacting with and inhibiting the anti-apoptotic function of Bcl-2 and Bcl-XL [82] and also, by sensitizing the cell to Bid-induced mitochondria disintegration [83]. Bad activity is determined by its hosphorylation status [82], which is modulated by protein kinases, including Akt [82] downstream growth factor receptor activation [67], allowing Bad dimerization and its sequestration away from the mitochondria [30]. As a response to death stimuli, Bad is dephosphorylated and translocated to the mitochondria to induce cell death [82]. In this regard, recent studies have shown that tumor suppressor p53 can strongly bind to dephosphorylated Bad, which determines Bad pro-apoptotic role [82]. The association between Bad and p53 also regulates Bad expression, by preventing p53 entrance into the nucleus to bind Bad promoter [82].
		Noxa (Damage in Latin)	BH3	Noxa is associated to the mitochondrial membrane [84] and can be activated, by exposing its BH3 domain and disrupting mitochondrial membrane [84] in response to cellular stress [77], such as DNA-damage [67], in a p53-dependent or -independent manner [77]. Noxa activation sensitizes the cell toward the action of other BH3-only proteins [77] and enhances the activation of Bax and/or Bak [77]. Noxa can also bind to anti-apoptotic members of Bcl-2 family [84], such as Mcl1, for proteasomal degradation [77], being also able to neutralize the pro-apoptotic effect of Bcl-XL [41].
		Harakiri (Hrk) DP5	BH3 TM	Hrk is associated to the mitochondria and is able to promote apoptosis via mitochondrial outer membrane permeabilization [85] as a response to different signals such as endoplasmic reticulum stress or cytokines [86]. Hrk is regulated by the interaction with the apoptotic inhibitors Bcl-2 and Bcl-X(L) via its BH3 domain [38,85] and its loss contributes to neoplasia and autoimmunity [85].
		Bcl-2-interacting killer (Bik)	BH3 TM	Bik localizes in the endoplasmic reticulum outer membrane [87]. As a response to stress signals and Bax activation, Bik promotes apoptosis mobilizing calcium to the mitochondria, remodeling the mitochondrial cristae [87] and provoking cytochrome c release [88]. BIK has been proposed as a new target for anti-cancer drugs that inhibit proteasomal functions as well as for the treatment of difficult cancers [87].

**Table 6:** Small molecules targeting Bcl-2-family proteins in clinical development [89].

	Type	Small Molecule Inhibitor	Disease
Phase I	Mcl-1 protein inhibitor	AMG-176 AZD-5991	Multiple myeloma
			Hematological cancer
		S-64315 (MIK-665)	Diffuse large B-cell lymphoma, multiple myeloma
		S-64315 (MIK-665)	Myelodysplastic syndrome, Acute myeloid leukemia (AML)
	Bcl-2 protein inhibitor	Venetoclax	Non-Hodgkin lymphoma, myelodysplastic syndrome
		BCL-201 (S-55746)	Mantle cell lymphoma, follicular lymphoma
		BCL-201 (S-55746)	Myelodysplastic syndrome, CLL, AML, NHL
	Bcl-2, Bcl-xL inhibitor	APG-1252	Tumor, small cell lung cancer
		AT-101	Multiple myeloma
		Navitoclax	Acute lymphoblastic leukemia
Phase II	Bcl-2 protein inhibitor	Venetoclax	Diffuse large B cell lymphoma, B cell lymphoma, myelodysplastic syndrome (suspended), follicular lymphoma
		Venetoclax	DLBCL, B cell lymphoma, myelodysplastic syndrome (suspended), Waldenström macroglobulinemia, hematological neoplasm, follicular lymphoma
	Bcl-2, Bcl-xL inhibitor	Navitoclax	Myelofibrosis
Phase III	Bcl-2 protein inhibitor	Venetoclax	Multiple myeloma, acute myeloid leukemia
		Venetoclax	Multiple myeloma, AML, mantle cell lymphoma
		Venetoclax	Chronic lymphocytic leukemia [31]

**Table 7:** Preclinical activities of BH3 mimetics [104].

Inhibitor	Cancer Models
HA14-1 and derivatives	Lymphoma, leukemia, myeloma, glioma, ovarian, prostate
BH3Is	Leukemia, cervical, glioma
2-Methoxy-antimycin A3	Lung, mesothelioma, esophageal
(-)-Gossypol	Lymphoma1, leukemia2, myeloma3, prostate, colon, head and neck squamous cell carcinoma
TW-37	Lymphoma, leukemia, prostate, lung, pancreatic, liver, nasopharyngeal
Apogossypolone and derivatives	Lymphoma, leukemia, prostate, lung, pancreatic, liver, nasopharyngeal
BI-97CI	Lymphoma, prostate, lung
Chelerythrine	Leukemia, liver, cardiac, neuroblastoma
YC137	Breast
Obatoclax	Lymphoma, leukemia, myeloma, lung, mammary carcinoma, colon, cervical, prostate
ABT-737/263	Lymphoma, leukemia, myeloma, prostate, lung, pancreatic, ovarian, colorectal, gastrointestinal
S1	Breast, liver, cervical
